# The Amending Bill in Committee

**Published:** 1913-08-09

**Authors:** 


### NATIONAL INSURANCE ACT DEPARTMENT.

### LXIII.—The Amending Bill in Committee.

After a singularly uninteresting debate on the second reading of the National Insurance Act Amendment Bill that measure was committed for consideration in Grand Committee as distinguished from a Committee of the whole House. All Bills of first-class importance are subject to detailed scrutiny, clause by clause, in the House itself, and an endeavour was made to obtain for the Amending Bill the advantages accruing from such full and open discussion of its proposals. The motion was put to the vote, but was lost by the substantial majority of 124.

One of the principal disadvantages of consideration in Grand Committee, as ordinarily conducted, is that the proceedings are not fully reported. So great an interest has been taken, however, in National Insurance, both in the House and among the public, that on the request of Mr. Ramsay Macdonald the Prime Minister agreed to the unusual course of having the proceedings officially reported. The Committee chosen to consider the Bill was that known as Standing Committee C., of which the Right Hon. J. W. Wilson is chairman. This Committee is a particularly strong one, and for the special purpose of this Bill it was strengthened by the addition of fifteen members of Parliament who have taken a special interest in the National Insurance Act. Mr. Booth, Mr. Cassel, Mr. Forster, Mr. Godfrey Locker-Lampson, and Mr. Worthington Evans—to mention five only of the gentlemen selected—were among those specially chosen, and it is clear from the part they took in the proceedings that the addition of experts to the Committee resulted in substantial advantage to the Bill as it emerged from the Committee stage of its Parliamentary course.

In view of the fact that the proceedings were reported day by day, coupled with the inclusion of expert members on the Committee, it now seems that the Bill received fuller consideration than would have been possible in a Committee of the whole House.

At the first sitting of the Committee the Chancellor of the Exchequer announced that it would be necessary to ask the Committee to make exceptional sacrifices in order to get through the work. To enable this to be done the Prime Minister moved a resolution in the House empowering the Committee to sit notwithstanding the fact that the House is sitting at the same time, thus following the precedent set when the unemployment insurance provisions of the Act were considered in Committee.

The daily reports of the proceedings testify to the assiduous manner in which the Committee addressed themselves to their task. Those who have followed the proceedings will be disposed to agree with the concluding remarks of Mr. Masterman, who was in charge of the Bill, when he said: "I thought it was a good Bill at the beginning, I think it is a much better Bill now, and I am glad that every party has co-operated in improving it."

### The Amendment Bill Amended.

The Bill "as amended by Standing Committee C" has now been published, and it would hardly be recognised as the same Bill as that which passed the second reading. As originally presented to Parliament the Bill contained but thirteen short clauses and one schedule. Several of these clauses have been considerably extended and other new clauses have been added, so that the Bill now consists of forty-one clauses and has two schedules.

As we have already in previous issues examined the original proposals of the amending Bill in some detail we do not propose to cover this ground again. It will be sufficient if we indicate the substance of the alterations that have been made to the original clauses and then pass on to an examination of the proposals contained in the new clauses that have been added. It has to be remembered that the Bill has yet to pass the report stage and third reading in the House of Commons before it finally goes to the House of Lords, and it is quite likely that further considerable alterations will be made before it becomes an Act of Parliament, short though the time now is before the conclusion of the Session.

### Alterations of the Original Clauses.

The consideration of the suggested amendments to the original clauses occupied much less time than the discussions on the new clauses. Clause 1, which is designed to give statutory authority for the additional grant made by the Chancellor of the Exchequer to the doctors for the administration of medical benefit, was passed as drafted. It was, however, freely criticised, for, as we took occasion to point out, the proposal is not limited to the specific purpose of regularising the grant that has already been made, but would confer the power of making additional Government grants in aid of National Insurance whenever these might be required.

The Chancellor of the Exchequer expressed the opinion that the restriction was unnecessary, and drew a parallel between the National Insurance Act and the Education Acts. He held that if it had been necessary for the Government of the day to have gone to Parliament to obtain legislative sanction for every grant in respect of free education, the cause would have suffered immensely. In the same way it is impossible to foresee at the present time, while national insurance against sickness is in its infancy, what additional charges may have to be met—it may be in emergency—and while disclaiming any intention of fundamentally altering the State contribution towards the benefits under the clause, he held that it was better to give the Government a free hand, as had been done in drafting the clause in question.

The second clause in the original Bill—now Clause 3—by which the reductions of benefit to persons over fifty years of age entering insurance as employed contributors are to be abolished was passed substantially as introduced. The plea which we voiced for the continuation of medical benefit to old persons, over sixty-five years of age at entry, until the end of life was admitted, and the only restriction now proposed is that at least twenty-six weekly contributions shall have been paid.

Old persons who were brought into insurance at a very advanced age and forced to contribute with little chance of obtaining any benefit for those contributions will now have no occasion to grumble at their lot, for in return for those contributions—so long as they are not less than twenty-six weeks—they will have free medical treatment and attendance for the remainder of life.

It may be remarked in passing that the clause as amended would appear to require revision on report, for as it now stands the abolition of the reduction of benefits only extends to those who entered insurance " within one year " of the commencement of the Act. By a new clause the benefits of the flat rate of contribution both for employed contributors and voluntary contributors, the latter up to the age of forty-five, are to be extended a further three months, making a total of sixy-five weeks as from the commencement of the Act. This is a drafting alteration which should not be overlooked.

No alteration of any description has been made to the clauses relating to the excuse of arrears, or that by which an income limit of £160 per annum is imposed, above which medical benefit cannot be obtained by voluntary contributors. The same remark applies to the proposal regarding sickness benefit when the insured person is in receipt of compensation. Exempted persons who are now, under the provisions of the Bill, to obtain medical and sanatorium benefits will have, by virtue of the amendments made in committee, to conform to the same income limit of £160 a year, but the regulations of the Insurance Commissioners may not require the payment of more than twenty-six contributions to entitle them to benefit.

Verbal alterations of no far reaching consequence are the only amendments that have been made to the remaining clauses of the original Bill.

### The New Clauses.

The first of the new clauses has already been alluded to above. It will give a further three months' grace for entry into insurance at full rates of benefit at ages above sixteen, and is a concession of value. By Clause 4, as the Bill now stands, persons who cease to be qualified for insurance as employed contributors and who have attained sixty years of age are to be entitled to remain insured as voluntary contributors. If, however, the cause of the disqualifications is that their income from all sources exceeds £160 per annum the proviso of the principal Act will still operate to exclude them from voluntary insurance unless they have been employed contributors for at least five years.

By Clause 5 it will be possible for a person to claim exemption from insurance in respect of employment otherwise bringing him within the compulsory provisions of the Act if he is able to show that he is " ordinarily and mainly dependent for his livelihood on the earnings derived by him from an occupation which is not employment within the meaning of the Act." An extension of the First Schedule to the Act whereby the employees of any local or other public authority, except those excluded by special order, are to become employed contributors is proposed by Clause 6.

Clause 8 would have the effect of repealing the provisions of the Act as regards the reduction of benefits when contributions are in arrear, and of substituting in their place reductions approximately equivalent to the value of the loss of contributions in accordance with a scale to be prescribed by the Insurance Commissioners. The object of this clause is apparently to enable the Commissioners to substitute half-yearly insurance cards in place of the present quarterly cards, and thus to reduce the work entailed on the approved societies and on the Commissioners in the collection of contributions with the advantage of saving in administrative cost.

The difficulties of construing Acts of Parliament were perhaps never better exemplified than in the new Clause 12. The object of this clause is to make the meaning of the principal Act quite clear in regard to the much debated question of no sick pay for the first three days of sickness. Instead of the words " commencing from the fourth day after being rendered incapable of work " it is proposed to substitute the words " commencing on the fourth day of incapacity." This is at first sight a great improvement, but the clause goes on to explain that " a day on which the incapacitated person was prevented by the incapacity from doing any *effective* work shall be treated as a day of incapacity." It does not require a legal training to foresee that the word *effective* if it is allowed to remain will cause endless trouble, and will for practical purposes reduce the safeguard of the first three days to two days only.

### Alterations in Maternity Benefit.

The longest discussion on any one subject by the Committee was that relating to maternity benefit. The result of that discussion is contained in Clause 13, by which the benefit is to be construed as a benefit for the " mother of the child," even though it be paid from the husband's insurance. Furthermore, instead of the payment of sickness benefit, for an uncertain period, out of her own insurance the woman who is herself an insured person is to be entitled to a maternity benefit, thus providing double maternity benefit as at present understood. There is, however, the qualification that the woman must abstain from remunerative work for four weeks after the confinement. It will be agreed that this amendment is in the right direction.

